# The impact of computer-based cognitive training intervention on the quality of life among elderly people: a randomized clinical trial

**DOI:** 10.1186/s13063-020-05008-4

**Published:** 2021-01-11

**Authors:** Leila kazazi, Mohsen Shati, Seyede Salehe Mortazavi, Vahid Nejati, Mahshid Foroughan

**Affiliations:** 1 Department of Aging, University of Social Welfare and Rehabilitation Sciences, Tehran, Iran; 2 Mental Health Research Center, Psychosocial Health Research Institute, Iran University of Medical Sciences (IUMS), Tehran, Iran; 3 School of Behavioral Sciences and Mental Health (Tehran Institute of Psychiatry), Iran University of Medical Sciences (IUMS), Tehran, Iran; 4 Spiritual Health Research Center, Iran University of Medical Sciences, Tehran, Iran; 5 Psychology Department, Shahid Beheshti University, Tehran, Iran

**Keywords:** Health-related quality of life, Aging, Cognitive training, Selective attention, Working memory

## Abstract

**Background:**

Through the process of normal aging, cognitive decline would cause a lower level of functioning in real life. This flow might interfere with health-related quality of life (QoL). The purpose of this study is to investigate the effect of computer-based cognitive intervention on increasing QoL of elderly people.

**Methods:**

A total number of 52 community-dwelling older adults participated in this study. This community scored ≥ 21 in the Mini-Mental State Examination (MMSE) and a clock drawing test score ≥ 4 from health centers in Tehran, Iran. This study is a parallel group stratified randomized clinical trial. The intervention group received a 45-min cognitive training session twice a week for 10 sessions, using Attentive Rehabilitation of Attention and Memory (ARAM) software focusing on selective attention and working memory. QoL was evaluated as a primary outcome. The control group participated in educational workshops.

**Results:**

From fifty-two persons, only one participant was excluded from the study in the intervention group during follow-up. Results revealed by increasing cognitive function, improvement occurred in QoL (*F* = 13.417, *p* value < 0.001, partial eta-squared = 0.324) as a primary outcome in the intervention group. Among eight domains of QoL, there was significant increasing in domain of role limitations due to emotional problems (*F* = 4.007, *p* value = 0.021, partial eta-squared = 0.059), social functioning (*F* = 2.423, *p* value = 0.044, partial eta-squared = 0.004), and role limitations due to physical health (*F* = 10.749, *p* value < 0.001, partial eta-squared = 0.026).

**Conclusions:**

Based on the results, ARAM showed transition and long-term effects on QoL in elderly people by improving cognitive functions such as selective attention and working memory.

**Trial registration:**

Iranian Registry of Clinical Trials IRCT2016122731602N1. Registered on June 21, 2017

## Background

In 2010, the global population aged 65 and older encompassed the total number of 524 million. This number, which contains about 8% of the population, is expected to increase by 1.5 billion until 2050 and also includes about 16% of the global population. The speed of this growth is higher in developing countries [1]. Applying these changes in population structure is due to consideration of how to preserve a high level of quality of life (QoL) and subsequently increase the life expectancy of older adults [2]. Health-related quality of life affects the health structure of an individual and the subjective evaluation of illness and treatment, which itself influences physical, mental, and social performance [3]. Age-associated cognitive decline or normal cognitive aging comes along with some deterioration in some mental domains such as processing speed, reasoning, memory, and executive functions, some of which lead to a decrease in general cognitive function [4]. Tucker-Drob showed that changes in neurocognitive function have a close relation with the ability of doing daily tasks in older adults’ living [5]. Age-related cognitive decline has a negative effect on QoL, independence, quality and quantity of social interactions, and participation in cognitive stimulator activities [6], but it is believed that individuals can improve their basic cognitive abilities through appropriate training. The ACTIVE study showed that through cognitive training, older adults aged 65–94 were able to make important achievements in processing speed, memory, and reasoning [7].

The aging population increase and the need to preserve QoL emphasize the necessity of rapid, effective, and affordable solutions to delay age-related cognitive decline [8]. Cognitive interventions which are used to improve cognitive domains are based on the neuroscience model of activities and chemical agents which improve neurodevelopment and even neural growth and plasticity [9]. It has been shown that the processes supported by the prefrontal cortex, such as attention, inhibition, and working memory, are sensitive to the decline associated with age [10]. Recent studies show that the relationship between working memory and selective attention is mutual and multidimensional [11] and probably controlled by common neural mechanisms. Working memory plays the role of visual control in selective attention [12], and selective attention itself is the main process of optimal performance in working memory [13, 14].

Considering age-related changes occurring in frontal lobes, the importance of transferring learned cognitive skills to outcomes such as QoL, and the lack of study about the relationship between cognitive function and quality of life (especially in older adults with age-related cognitive decline), the purpose of this study was to evaluate the effectiveness of cognitive training in improving working memory and selective attention and subsequently improving QoL in older adults with normal cognitive function (which had shown age-related cognitive decline in some aspects of cognition). Since computer-based cognitive training intervention is more challengeable with visual appeal, gradation ability, high-quality assignments, and the ability to adapt to individual performance [15], computer-based cognitive training intervention was used in this study.

## Methods

### Study design

This study has been designed based on the parallel group stratified randomized clinical trial. The failure to blinding of participants (according to the nature of the intervention) and increasing the follow-up duration (3 months) was changed to study design after registering the clinical trial and start of the intervention.

### Participants

The subjects were older adults aged 60 years and above from the general population who were attended in health centers of the municipality in west of Tehran. These centers have been established mostly to increase social participation and recreational activities of older adults and also to check their blood pressure and glucose levels as routine services. In order to determine the confounder variable in the first phase, sampling was performed in two regions from each area of Tehran (north, west, east, center, and south). On the other hand and due to accessibility, participants for the clinical trial were selected from three health centers in west of Tehran. The subjects who were obtained an MMSE score > 21 and a clock drawing test score ≥ 4 (concerning inclusion criteria) in the previous phase were invited to participate in the clinical trial by phone call. The screening of participants for the first stage was published elsewhere [16]. The participants who entered the intervention were consented for screening and met the above criteria. Baseline parameters were evaluated between August 2016 and October 2016 which was 7 months before intervention in phase Ӏ. The intervention period was between June 5 and July 21, 2017, and the follow-up period was between July 21 and October 21 (3 months following the last session) in both groups.

### Eligibility criteria

Eligible participants were older adults aged 60 years and above who were able to communicate in Persian. According to the Persian validated version of Mini-Mental State Examination (MMSE) test, at least 4 years of education is necessary to respond to the items of the test [17]. Eligible participants achieved a MMSE score > 21 (cut point for Iranian elderlies to rule out dementia) [17] and a clock drawing test score ≥ 4 (cut point based on Shulman’s rating for Iranian elderlies to rule out dementia) [18].

Exclusion criteria contained diagnoses of a disease which could lead to extreme cognitive or functional decline. The instances are a stroke throughout the previous 12 months, end-stage cancer, dementia and Parkinson disease, and uncorrected visual or auditory impairment. Individuals with subjective complaints of memory impairment also were excluded.

### Intervention

The intervention group attended 12 sessions of a cognitive intervention schedule twice a week. The first and last sessions were dedicated to evaluation and 10 sessions to intervention. The 45-min sessions implemented the Attentive Rehabilitation of Attention and the Memory (ARAM) software application. ARAM is part of Neurocognitive Joyful Attentive Training Intervention that has been designed as a tool for cognitive rehabilitation. The grading system depends on the number of equivocal stimuli, speed of exhibition of stimuli, number of goals for stimuli, and changing task rules. The program consists of 10 graded progressive tasks. All tasks have 10 levels, and participants could shift to the higher level after gaining the 80% of the score in each level. The ARAM tasks are designed based on the hierarchical model of attention [19] and Baddeley’s model of working memory as following [20].

### Preparing task

In this task, the subject focuses his attention on one imminent stimulus. There exists no unrelated stimulus at this phase.

### Search and selection task

In this task, the target stimulus is defined for the subject and other stimuli appear as disturbing ones. The score of this task is calculated based on the speed and accuracy of the subject’s responses.

### Maintenance task

The maintenance task is the ability to allocate attention to a stimulus source a long time after the emergence of the stimulus.

### Transfer and inhibitory task

Throughout this task, the individual arranges a set of stimuli based on a variable rule. The ability to transfer from one rule to another is reinforced in this task.

These tasks are presenting as follows:

1. Colored home task. A schematic simple home, with different colors of roof, wall, windows, and door, appears as a target on the top of the page. The participant was instructed to find out the target home in one cell of the table with an almost similar image in each cell. Participants should respond as fast and accurate as possible. The contrast of colors, the number of distractors, and the variety of sample homes in each trail are used for increasing the difficulty of the task. This task is designed for the training of sustained and selective attention.
2. Face task. Some faces falling down on the top of the screen in different points, moving with arrow keys. The faces were different in some features which consist of hair color (black, brown, and gray), skin color (white, brown, and black), and emotional expression (sad, happy, and neutral). The participants should arrange the faces on top of each other with respect to the given rule. There were three changing rules which consist of skin color, hair color, and emotion of faces. Three correct matchings have a score. The speed of falling faces and changing rule increased across progression. This task engages sustained, selective, shifting, and divided attention to perform.
3. Similar window task. In this task, a table appears on the screen within some similar hidden images. Clicking on each cell discovers the hidden image until the next click. If two similar cells are clicked in a row, they would remain open. The instruction is matching similar images in different cells of the table. The number of chains that should be clicked in raw, number of cells, similarity, and meaning of shapes are used for increasing the difficulty of the task. This task trains the visuospatial span in working memory.
4. Marked tables’ task. In this task, a sequence of cued tables, marked in one cell, appear on the screen in a row with a predefined show time and inter-stimulus interval. Then, after a while, four tables appear on consist of different similar cues in cells as choices. The participants were instructed to choose the table with respect to the spatial location of cues in the trail. The number of single cued tables, show time, inter-stimulus interval, and delay of choices are used for difficulty. This task trains the visuospatial span in working memory.
5. Segmented image task. In this task, different fragments of an image are presented serially and after a delay the whole image should be selected between four choices. The number of fragment, inter-fragment intervals, delay of choices, complexity of image, and similarity of choices are used for increasing the difficulty of the task. This task trains the visuospatial span component of working memory.
6. Acronym making task. Different words appear on the screen for a given time serially, and after a delay, four words appear as choices. The trainee is instructed to make a word from the first letter of the stimuli word and find it in the choices. The number, meaning, and length of stimuli as well as similarity of choices are used for increasing the complexity of the task. This task serves phonological processing, inhibitory control, and phonological span to perform.
7. Last colored task. In this task, a sequence of colored squares appeared on the screen serially, and after that, 4 choices appear to select. Each choice consists of a list of two colored cells. The participant should select the choice based on the last color in the order. The variety of colors, sequence items, and number of choice items are used for increasing the difficulty of the task. This task is used for training updating ability.
8. Animal tracing task. A table that consists of an animal in one cell appears on the screen for a limited time. Then, a sequence of arrows appears serially with a predefined show time and inter-stimulus intervals. The participants are instructed that each arrow means movement of an animal to the corresponding neighbor cells. After the sequence of the arrow, they should say about the new location of the animal based on the given choices. The number of table’s cell, number of arrow, and variety of direction are used for progression of the complexity of the task. This task trains the visuospatial span and updating component of working memory.
9. Repetitive image task. In this task, a series of different images appear on the screen that some of them were repetitive. The participant had to point the repetitive images. The goal percent, complexity, and similarity of images are used for the progression of the task. This task based on N-back paradigm improves the updating abilities.
10. Letter matching task. In this task, a series of words or sentences is presented serially and the participant should reply whether the initial letter of the current text is similar to the last letter of the previous text or not. The length of the text is used for increasing the complexity of the task. This task engages the phonological span for the holding of target letter and inhibition and updating for inhibition of unwanted part of the text. These tasks are modified in terms of difficulty, speed, and number [21].

In this research, each task was performed orderly in each session and was graded according to the subject’s progress. The effectiveness of ARAM in improvement of attention and working memory found in including improved attention and active memory in adolescents with leukemia [22], enhanced working memory and reading components in students with dyslexia [23], improved behavioral syndrome [24], executive performance in children with attention-deficit and hyperactivity disorder [25], enhanced attention in children with developmental stuttering [26], and enhanced executive performance in the elderly [27].

### Control group

The participants who were assigned to the control group have participated in educational workshops concerning age-related cognitive changes and cognitive disorders. Participants have been acquainted with age-related memory changes, health and lifestyle factors that affect memory, applied strategies to optimize memory function, strategies for older adults with normal age-related memory changes, signs of Alzheimer, and understanding Alzheimer’s and dementia. These educational workshops occurred in group sessions and were held 5 sessions once a week.

### Outcomes

Quality of life was the primary outcome of the study and was assessed before and 3 months after the intervention in the intervention group. Assessments in the control group were performed simultaneously under the same conditions and by the same observer.

The Iranian version of the Short Form (SF-36) health questionnaire was used to assess QoL [28, 29]. This questionnaire assessed eight health concepts: physical functioning, role limitations due to physical health, pain, general health, energy/fatigue, social functioning, role limitations due to emotional problems, and emotional well-being [30].

We aimed to assess health status in order to determine the effectiveness of the intervention. The high scores of the (SF-36) health questionnaire demonstrate better physical and mental health condition.

The secondary outcome measure was the cognitive function. It was measured basically by the Iranian version of MMSE [17]. The Mini-Mental State Exam (MMSE) is a widely used test of cognitive function among the elderly; it includes tests of orientation, attention, memory, language, and visual-spatial skills. A decline in cognitive function in turn could adversely impact the physical functioning and quality of life of older adults [31].

The specific domain of the cognitive function was assessed by the Wisconsin card sorting test, the Stroop test, the N back test, and the go/no go test.

The Wisconsin card sorting test, which is a major indicator of the activity of the prefrontal cortex, developed by Grant and Berg in 1948, was used to assess problem-solving and decision-making skills [32]. The Stroop test presented by Macleod in 1991 was used to measure the performance of the prefrontal cortex and selective attention. The N back test, introduced by Wayne Kirchner in 1958, was used to assess working memory. The go/no go test was an indicator of inhibitory control, in which two types of situations of “go” and “no go” were randomly assigned to one task and the ability of an individual to control his response in the second situation [33]. These tools were used to assess the different aspects of working memory and selective attention before and after the implementation of the intervention. There were no changes in study outcomes after starting the trial.

### Sample size

By using G-Power software (version 3.1.9.2) [22] for comparing two independent means, because in a previous study it was considerably large effect size (*d* = 0.8) with respect to alpha 0.05 and beta 0.2, the sample size of this study was calculated about 52 (*α* = 0.05).

### Recruitment/enrollment

The number of 91 older adults was assessed for eligibility; about 5 participants did not meet inclusion/exclusion criteria, and 34 participants declined to participate in the cognitive rehabilitation program; therefore, 52 participants were assigned to two groups by the health center’s secretary using balance blocked randomization. These two groups consisted of 26 participants in the intervention group and 26 participants in the control group. The randomization table was only available to the secretary of the health center who enrolled participants and assigned them to the study groups.

### Randomization

In the previous study, two variables of educational level and depression could be considered as potential confounder in relation to cognitive function and QoL [16]. Accordingly, in this study, a stratified randomized design was decided to be used.

**Fig. 1:**
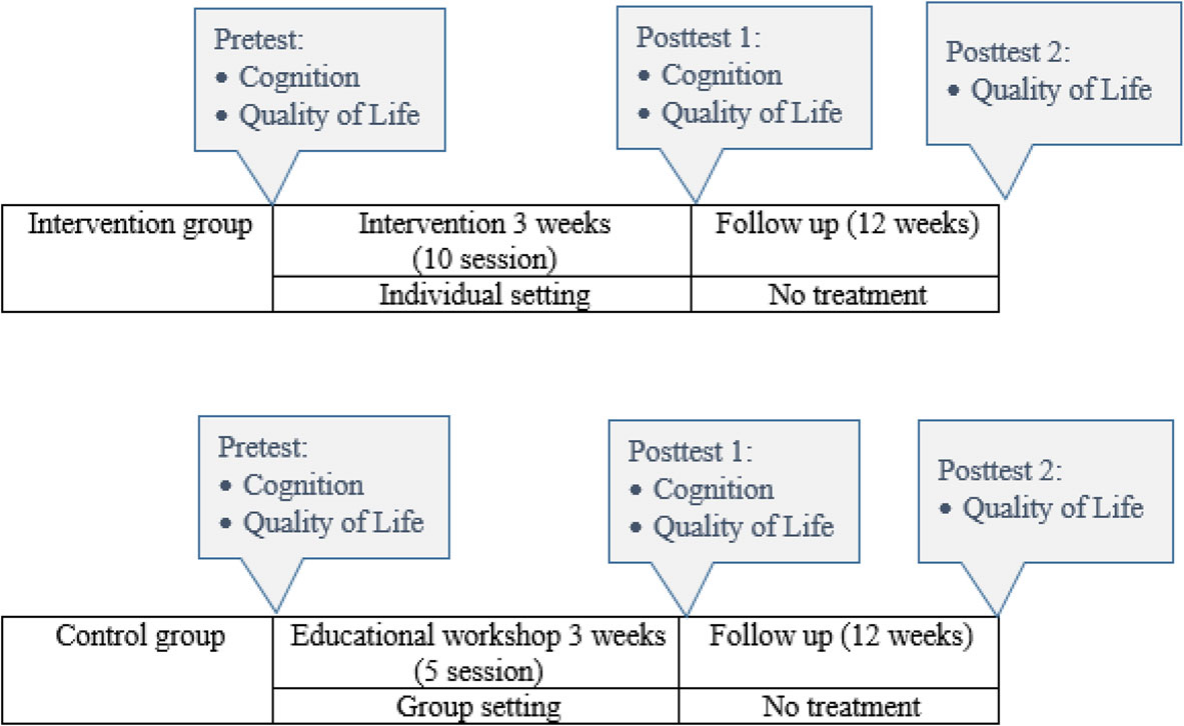
Time point schedule of the randomized clinical trial and the prospective follow-up

At the first phase of the study, educational level (*B* = 2.704; 95% CI 2.09 to 3.30; *p* <  0.001) and depression (*B* = 2.554; 95% CI 2.00 to 3.10; *p* <  0.001) were considered as two potential confounding variables. After adjusting for potential confounders in the regression model between QoL and cognitive function, 87% of older adults in the previous study was depressed due to GDS assessment and the educational level of 26.4% was elementary school and below [16]. Thus, a stratified randomized design was implemented to control the potentially confounding effect of these two binaries. Four randomizing sequences were used (the combination of depression and low educational level, depression and high educational level, high educational level without depression, and low educational level without depression), and the participants were divided into either the intervention or control group based on these different strata. The expert epidemiologist generated the random allocation sequence and a gerontologist who was trained in ARAM software application implemented intervention and workshop sessions. During the clinical trial, one participant was excluded from the study because of having surgery in the intervention group (data of this participant was imputed as missing data); nevertheless, in the control group and in the follow-up procedure, no exclusion occurred (Fig. 1). Finally, pulled data was analyzed. The analysis was performed by originally assigned groups and there was no deviation in the protocol of the study. The intervention did not require a facilitator.

### Blinding

According to the nature of the intervention, the participants could not be blinded in the process of intervention; also, the assessors/observers were not masked. Only the person who analyzed the data was blinded to the allocation into the intervention or control groups.

### Statistical analysis

All participants who were randomized were included in the statistical analysis and analyzed according to the group they were originally assigned. All statistical analyses were done by using SPSS version 18. Comparison of QoL as a primary outcome in the control and intervention groups in each assessment (before training, after training, and follow-up) was made by using the repeated measure analysis of variances. The two-tailed *t* test was used to compare the cognitive functioning of the older adults in the control and intervention groups. There was no statistical analysis plan. A *p* value of less than 0.05 was considered to be significant.

## Results

**Fig. 2:**
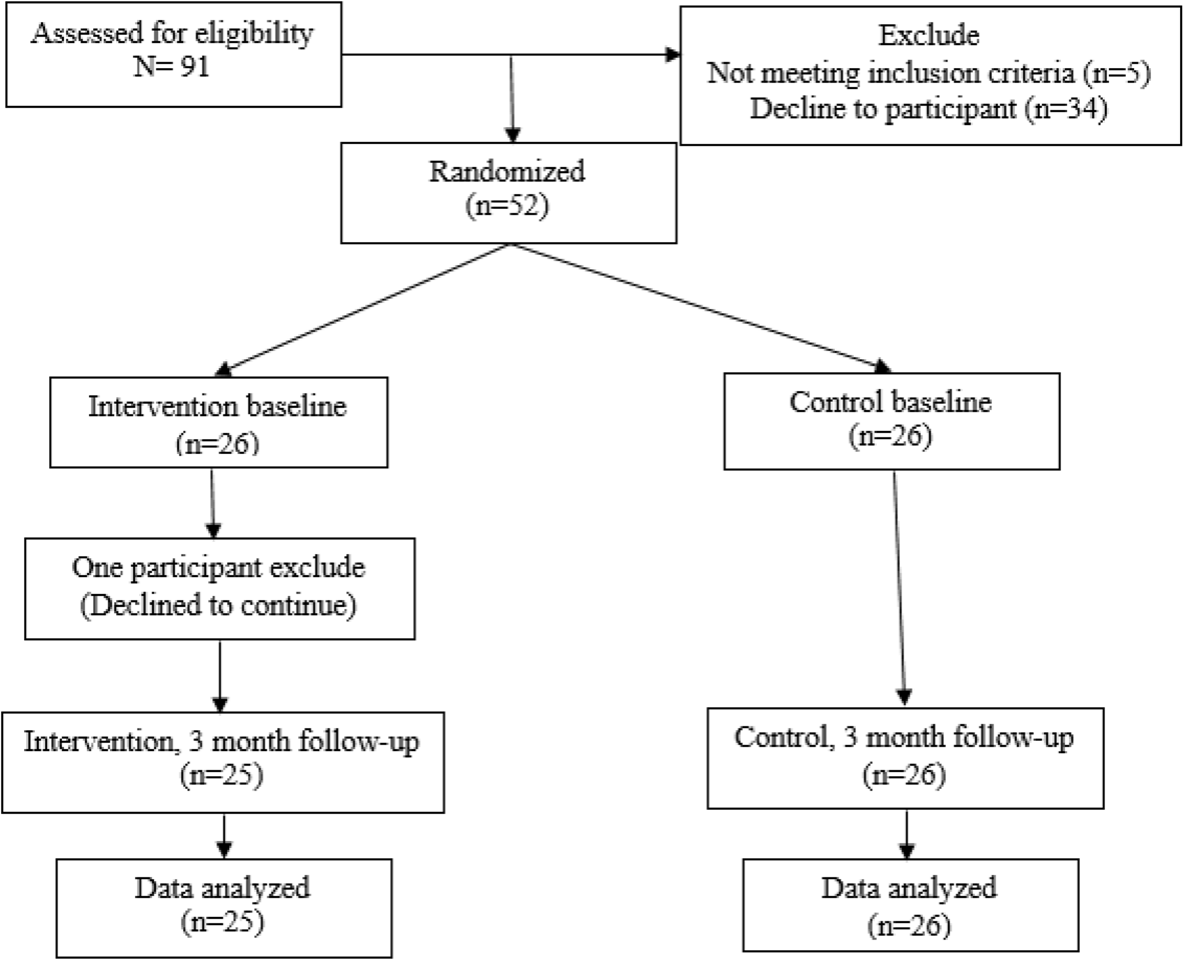
Overview of participants’ flow

The trial enrolled 91 older adults, from whom 52 were randomly assigned to intervention and control groups (Fig. 2). The average age was almost similar in the intervention and control groups. About half of the participants in each group were women. Most of the participants were married and academically educated (Table 1).

**Table 1:** Participants’ demographic characteristics

Variables	Intervention group (***n*** = 26)	Control group (***n*** = 26)
**Age (year) (mean** ± **SD)**	65.42 ± 5.40	64.38 ± 5.00
**Gender (women) (*n*) (%)**	15 (57.7)	13 (50.0)
**Marital status** ***n*** **(%)**		
Married	21 (80.7)	23 (88.4)
Single	2 (7.6)	1 (3.8)
Widow	3 (11.5)	2 (7.6)
**Education (*n*) (%)**		
High school degree	2 (7.6)	3 (11.5)
High school diploma	13 (50.0)	13 (50.0)
Associate degree	2 (7.6)	3 (11.5)
Bachelor degree and more	9 (34.6)	7 (26.9)

As shown in Table 2, the changes in the MMSE before and after the intervention were significant in the intervention group in comparison with the control group (mean = 0.46 (1.50), *p* value = 0.006, 95% CI − 1.80, − 0.31).

**Table 2:** Comparing general and specific domains of cognition before/after the intervention (two-tailed *t* test)

Cognitive assessment			Intervention group, mean (SD)			Control group, mean (SD)			***p*** value	95% CI of between groups difference of differences
			Before	After	Before–after difference	Before	After	Before–after difference		
**MMSE**			26.81 (1.89)	28.28 (1.79)	1.52 (1.12)	27.04 (1.77)	27.50 (2.08)	0.46 (1.50)	0.006	(− 1.80, − 0.31)
**N-Back1**	True answer		17.15 (5.99)	20.48 (4.94)	3.52 (4.94)	19.38 (6.74)	19.62 (7.46)	0.23 (6.27)	0.043	(− 6.47, − 0.10)
	Total time		290.68 (143.36)	237.81 (106.17)	− 54.64 (101.11)	235.93 (91.59)	200.30 (65.33)	− 35.63 (58.72)	0.419	(− 27.29, 65.31)
**N-Back2**	True answer		11.08 (5.73)	18.68 (5.16)	7. 60 (6.71)	13.19 (6.48)	12.96 (6.35)	− 0.23 (8.12)	< 0.001	(− 12.03, − 3.62)
	Total time		288.32 (153.78)	198.73 (104.75)	− 90.05 (99.45)	277.12 (124.59)	299.11 (96.33)	− 48.01 (76.74)	0.099	(− 7.83, 91.91)
**WCST**	True clusters		3.23 1.42	4.60 (0.50)	1.40 (1.04)	3.23 (1.03)	3.65 (1.23)	0.42 (1.17)	0.003	(− 1.60, − 0.35)
	Preservation error		14.85 (6.23)	7.28 (2.50)	− 7.52 (6.07)	14.31 (5.67)	11.00 (5.38)	− 3.30 (7.03)	0.026	(0.50, 7.91)
	Total time		470.39 (162.41)	342.34 (96.63)	− 130.70 (110.19)	418.06 (121.58)	384.60 (113.77)	− 33.46 (108.23)	0.003	(35.76, 158.70)
**Go/no go**	Accuracy of performance stage		20.73 (3.94)	22.44 (2.31)	1.80 (2.95)	21.88 (2.30)	22.35 (2.57)	0.46 (1.83)	0.047	(− 2.73, 0.06)
	Accuracy of inhibitory stage		− 1.96 (7.19)	2.88 (2.66)	4.84 (6.16)	5.62 (11.70)	7.19 (8.30)	1.57 (5.89)	0.049	(− 6.65, 0.12)
**Stroop**	Color stage	Accuracy	98.38 (3.57)	99.36 (1.89)	1.04 (3.70)	98.92 (2.13)	98.00 (3.62)	− 0.92 (3.80)	0.048	(− 4.07, 0.15)
		Speed	1.45 (0.43)	1.29 (0.36)	− 0.17 (0.26)	1.32 (0.32)	1.39 (0.39)	0.06 (0.33)	0.007	(0.06, 0.40)
	**Color–word stage**	Accuracy	98.38 (4.00)	99.92 (0.40)	1.60 (3.82)	98.77 (2.47)	98.77 (2.19)	0.00 (2.99)	0.104	(− 3.52, 0.32)
		Speed	4.64 (17.00)	1.16 (0.31)	− 3.61 (17.39)	1.19 (0.27)	1.300 (0.31)	0.10 (0.17)	0.280	(− 3.45, 10.90)
	Interference stage	Accuracy	80.31 (23.73)	96.32 (4.88)	16.64 (23.65)	84.85 (25.03)	80.54 (30.70)	− 4.30 (35.18)	0.016	(− 37.88, − 4.00)
		Speed	2.41 (0.90)	1.79 (0.42)	− 0.62 (0.88)	2.42 (0.75)	2.10 (1.20)	− 0.14 (0.85)	0.044	(− 0.008, 0.96)

*MMSE* Mini-Mental State Examination, *WCST* Wisconsin card sorting test

Domain-specific cognitive assessment showed that there is a significant difference between the intervention and control groups in working memory score [(NBack1, total time: mean = − 35.63 (58.72), *p* value = 0.419, 95% CI − 27.29, 65.31) (NBack2, total time: mean = − 0.23 (8.12), *p* value < 0.001, 95% CI − 12.03, − 3.62) (WCST, true clusters: mean = 0.42 (1.17), *p* value = 0.003, 95% CI 0–1.60, − 0.35) (WCST, preservation error: mean = − 3.30 (7.03), *p* value = 0.026, 95% CI 0.50, 7.91)] and selective attention score [(go/no go, accuracy of inhibitory stage: mean = 1.57 (5.89), *p* value = 0.049, 95% CI − 6.65, 0.12) (Stroop, interference stage: accuracy mean = − 4.30 (35.18), *p* value = 0.016, 95% CI − 37.88, − 4.00; speed mean = − 0.104 (0.85), *p* value = 0.044, 95% CI − 0.008, 0.96)] and the repeated measure method was used to evaluate the changes over time in the QoL variable and its dimensions from before intervention to follow-up time in both groups. Regarding the *p* value, the sphericity hypothesis for variables of QoL and dimensions of role limitation due to emotional problems, energy/fatigue, emotional well-being, social function, and pain were established. Sphericity is an important assumption of a repeated measure ANOVA. It is the condition where the variances of the differences between all possible pairs of within-subject conditions are equal. Mauchly’s test of sphericity evaluates whether the sphericity assumption has been violated. In variables of physical functioning, role limitation due to physical function and general health, as the homogeneity of variance–covariance matrix hypothesis was not established, so the Greenhouse–Geisser alternative test was used in these cases. The Greenhouse–Geisser correction is a statistical method of adjusting for lack of sphericity in a repeated measure ANOVA. Accepting the Mauchly hypothesis, there was a significant difference between groups in the process of changes in QoL (*F* = 13.417, *p* value < 0.001, partial eta-squared = 0.324), role limitations due to emotional problems (*F* = 4.007, *p* value = 0.021, partial eta-squared = 0.059), and social functioning (*F* = 2.423, *p* value = 0.044, partial eta-squared = 0.004). According to the Greenhouse–Geisser test, there was a significant difference between intervention and control groups in role limitations due to physical health (*F* = 10.749, *p* value < 0.001, partial eta-squared = 0.026) (Table 3).

**Table 3:** Before intervention, after intervention, and follow-up QoL score among older adults (repeated measure analysis of variances)

Variable	Intervention (mean + SD)	Control (mean + SD)	***F***	***p*** value	Effect size (partial eta-squared)
QoL (before)	62.318 (14.972)	67.546 (18.335)	13.417	< 0.001	0.324
QoL (after)	70.775 (15.491)	65.708 (17.962)			
QoL (follow-up)	71.171 (16.234)	65.577 (18.231)			
RE (before)	56.057 (36.203)	61.535 (36.138)	4.007	0.021	0.117
RE (after)	77.270 (33.153)	58.971 (34.394)			
RE (follow-up)	78.786 (31.783)	60.253 (32.687)			
E/F (before)	61.363 (12.457)	63.846 (17.047)	0.076	0.927	0.004
E/F (after)	63.318 (15.655)	64.230 (16.834)			
E/F (follow-up)	61.363 (15.132)	62.480 (23.488)			
EW (before)	70.727 (12.306)	69.192 (19.312)	0.035	0.966	0.002
EW (after)	71.454 (16.621)	68.769 (16.895)			
EW (follow-up)	71.181 (13.810)	68.692 (18.196)			
SF (before)	73.863 (25.851)	80.288 (18.087)	2.423	0.044	0.095
SF (after)	82.288 (22.380)	74.038 (23.163)			
SF (follow-up)	84.659 (15.397)	79.326 (24.223)			
P (before)	58.590 (28.254)	65.942 (25.374)	0.581	0.561	0.026
P (after)	63.636 (24.540)	62.980 (29.664)			
P (follow-up)	69.772 (19.592)	69.711 (31.775)			
PF (before)	70.681 (20.947)	77.970 (18.197)	1.558	0.219	0.059
PF (after)	74.772 (21.071)	77.393 (17.645)			
PF (follow-up)	73.409 (25.042)	71.645 (22.991)			
RP (before)	45.075 (36.429)	68.269 32.831	10.749	< 0.001	0.264
RP (after)	79.545 (31.468)	60.192 (35.650)			
RP (follow-up)	76.136 (34.912)	56.512 (41.328)			
GH (before)	51.318 (15.465)	55.192 (18.026)	0.905	0.394	0.032
GH (after)	57.318 (11.917)	56.923 (16.617)			
GH (follow-up)	57.215 (13.012)	55.961 (14.630)			

*QoL* quality of life, *RE* role limitations due to emotional problems, *E/F* energy/fatigue, *EW* emotional well-being, *SF* social function, *P* pain, *PF* physical function, *RP* role limitations due to physical health, *GH* general health

## Discussion

The present study aimed in evaluating the effectiveness of computer-based cognitive intervention on improving the cognitive function in elderly people with normal cognitive aging and also transferring these changes to QoL as an outcome. Many research has proven that good functional ability is relevant to perceive good health and quality of life at older age, while only a few studies have evaluated the importance of cognitive function on mental health for HRQoL perception [34].

The results of the study indicate that the computer-based cognitive training had an effect on cognitive function and QoL and its dimensions. Among different dimensions of QoL, there was no difference between groups in dimensions of energy/fatigue, pain, physical function, emotional well-being and general health; however, the overall score of QoL showed a significant difference.

There was no significant improvement in the physical and general health domains of QoL. Generic quality-of-life instruments are not as sensitive as disease-specific questionnaires that focus on specific aspects of health problems and can be responsive to small but important changes in health [35].

This result indicates that the intervention improved cognitive performance and consequently the QoL of the individuals. The persistency of intervention effect on QoL was observed after the 3-month follow-up test. Hwang, Lim, and Lee analyzed the factors influencing life satisfaction based on the cognitive function level of normal elderly people over 60 without a diagnosis of dementia. Their study revealed that there is an increase in depression and lowered quality of life corresponding to lowered cognitive levels in the elderly [36].

There are several potential arguments for the effectiveness of our cognitive training. First of all and based on the available resources, cognitive training is effective when the participants face challenging, but not tiring, tasks. Secondly, individuals participate in tasks in order to remove their cognitive limitations [37].

One of the strengths of this study was the continuation of intervention effectiveness in QoL after 3 months of follow-up. Most recent studies on the cognitive change caused by natural aging have shown that attention and working memory are negatively affected by aging; thus, damage to these cognitive functions can have a profound effect on the QoL of older adults [11].

In the transfer model, the relationship between the level of cognitive function and QoL is considered to be an inter-level or vertical transfer. In the proposed model for brain–behavior interaction, transition is defined in three levels of hierarchy: brain level, cognitive level, and behavioral level. The activities of these levels are linked. The transfer model is like a pyramid with the brain at the top and behavior at the bottom. Transmission is a mutual process between the brain and behavior that modulates cognitive function. In the pyramid transfer, each behavior is supported by distinct cognitive domains. Intervention at the cognitive level is more flexible and transferable. In other words, a top–down transfer is stronger than a bottom–up transfer [21]. By this justification, in the top–down transfer model, by enhancing working memory and selective attention, an improvement was obtained in the QoL.

ARAM software has been used by Nejati et al. in order to improve the cognitive functioning of healthy older adults. Although significant changes in executive functions such as attention control, attention maintenance, inhibitory control, and cognition transfer were observed, the ability to maintain and transfer these skills was not evaluated [27].

The cognitive training protocol in this study was able to influence the working memory and selective attention of the older adults participating in the cognitive training program and improved cognitive performance. In a meta-analysis study, 17 studies were reviewed and there was a significant change in episodic memory, executive function, and working memory in patients with mild cognitive impairment in comparison to a healthy control group [38].

Finally, our results revealed that although a healthy body plays an important role in enhancing the quality of life, increasing cognitive function acts as factors with the greatest influence on the HRQoL in the elderly. In keeping with this result, establishing strategies to improve QoL is necessary. These plans consist of improving cognitive function, physical function early detection and intervention to enhance the HRQoL of elderly people. For generalization and extrapolating the result of the study to another set of participants, it is recommended that the intervention be applied in a greater sample size and a different setting (Alzheimer disease, mild cognitive impairment) with longer follow-up duration.

### Research limitations

One of the limitations of this study is that 10 sessions of cognitive training and also 3-month duration of follow-up may not be enough to show transfer effects efficiently, so increasing the number of intervention sessions and duration of intervention in follow-up periods is suggested for future studies. Another limitation was the lack of blinding for participants.

## Conclusion

This research is one of the first clinical trials conducted in Iran which specifically focuses on improving cognitive functioning in elderlies with age-related cognitive decline. By considering the results of this study, the enhancement of specific cognitive domains (selective attention and working memory) could enhance overall cognition.

## Data Availability

On completion of this study, the dataset will be made available from the corresponding author on reasonable request.

## Abbreviations

QoL: Quality of life
MMSE: Mini-Mental State Examination
ARAM: Attentive Rehabilitation of Attention and Memory
